# 

**Published:** 1843-09

**Authors:** 


### CONTENTS

OF NO. III.

---

### ORIGINAL COMMUNICATIONS.

ESSAYS AND CASES.

ART. I.—Aneurism of the Common Carotid Artery, being an Analysis of Seventeen Cases of this disease. By ROBERT S. WENDEL, M.D., of Murfreesboro, Tennessee. 161
ART. II.—A description of the Summer and Autumnal Diseases as they occur in the Wabash Valley, and particularly in Posey County, Indiana, and in White County, Illinois. By ROBERT ROBSON, M.D., of New Harmony, Indiana. - - - - - - 170

SELECTIONS FROM AMERICAN AND FOREIGN JOURNALS.

Clinical and Pathological Report on the Pneumonia of Children as it Prevails among the Poor in London. By CHARLES WEST, M.D., Physician to the Royal Infirmary for Children, and to the Finsbury Dispensary. - - - 184
Poisoning by Arsenous Acid ultimately detected by Examination of the Liver. - - - - - 220
Onyxis—the nail grown into the flesh. - - - 231
Dr. M'Cormac on Prolapsus Ani. - - - - 231
Antidote for the Bichloride of Mercury. - - - 232

The Botanical Labours of Mutis. - - - - - 232
Edinburgh Professors. - - - - - - 233
New Astringent Preparation. - - - - - 234

ORIGINAL INTELLIGENCE.

Travelling Editorials. - - - - - 236
Travelling Editorials and Travelling Editors. - - 240

